# Implementation of Health Empowerment Theory-Based Personalized Health Promotion in Village Health Volunteer Risk Group for Non-Communicable Diseases: A Mixed-Methods Study

**DOI:** 10.3390/healthcare14081006

**Published:** 2026-04-11

**Authors:** Supansa Srikong, Patcharin Phooncharoen, Suranun Klinsrisuk, Jakarin Thapsaeng, Wichai Eungpinichpong, Le Ke Nghiep, Kukiat Tudpor

**Affiliations:** 1 Department of Physical Therapy, Maha Sarakham Municipality Medical Center, Maha Sarakham 44000, Thailand; kwang_pttu@hotmail.com; 2 Department of Physical Therapy, Wapipathum Hospital, Maha Sarakham 44120, Thailand; onyourmn.nie@gmail.com; 3 Department of Physical Therapy, Primary Healthcare Service, Maha Sarakham Hospital, Maha Sarakham 44000, Thailand; klinsrisuk@gmail.com (S.K.); pt.pccmskh@gmail.com (J.T.); 4 School of Physical Therapy, Faculty of Associated Medical Sciences, Khon Kaen University, Khon Kaen 40002, Thailand; wiceun@kku.ac.th; 5 Vinh Long Department of Health, Vinh Long 85000, Vietnam; lekenghiep@gmail.com; 6 Public Health and Environmental Policy in Southeast Asia Research Cluster (PHEP-SEA), Mahasarakham University, Maha Sarakham 44150, Thailand; 7 Faculty of Public Health, Mahasarakham University, Maha Sarakham 44150, Thailand

**Keywords:** village health volunteers, non-communicable disease, health empowerment theory, bioelectrical impedance analysis, mixed-methods study

## Abstract

**Objective:** Village Health Volunteers (VHVs) are vital to Thailand’s primary healthcare, yet many face high risks for non-communicable diseases (NCDs). This preliminary study aimed to implement health empowerment theory-based personalized health promotion for individuals in the NCD-risk group. **Methods:** The preliminary mixed-methods study implemented a 6-month empowerment-based health promotion program for 21 VHV leaders (mean age 62.43 ± 7.28 years) at risk for NCDs. The intervention integrated laboratory data, behavioral and qualitative focus-group insights, and quantitative anthropometric data obtained via bioelectrical impedance analysis (BIA). **Results:** Participants’ exercise adequacy significantly improved after the intervention, increasing from 8.3% to 61.9% (*p* = 0.03). BIA revealed a physiological shift toward improved energy homeostasis, including decreased body weight, reduced visceral fat area, and increased muscle hydration. While biochemical markers did not reach statistical significance, clinically favorable downward trends were observed in median HbA1c (8.0% to 7.3%) and LDL cholesterol (141.8 to 119.0 mg/dL), alongside stable renal and liver function. Qualitative thematic analysis identified four primary domains of impact: sustainability and systemic advocacy, personal transformation, broad competence acquisition, and enhanced social capital. Participants reported a marked increase in self-efficacy, transitioning from inactive beneficiaries to active health advocates. This change was largely driven by mastery experiences, such as visible improvements in body composition and functional health literacy. **Conclusions:** The empowerment program significantly improved physical activity and body composition while fostering the social capital and health literacy necessary for community leadership, suggesting that personal health mastery is a critical precursor to effective systemic advocacy and long-term sustainability in community-led health programs.

## 1. Introduction

Non-communicable diseases (NCDs) represent the most significant public health challenge in Thailand, accounting for 76% of all national fatalities [1,2]. Addressing this epidemic requires moving beyond “one-size-fits-all” approaches toward personalized healthcare management tailored to an individual’s specific context [3]. In the Thai health system, the success of such personalized strategies relies heavily on Village Health Volunteers (VHVs) as the primary bridge between formal systems and the community [4,5].

Current literature extensively documents that the effectiveness of VHV or community health worker (CHW) interventions is linked to systemic empowerment—defined as a volunteer’s confidence and perceived control within their professional role [6,7]. However, a critical research gap persists regarding the distinction between professional empowerment for community service and personal empowerment for self-care. While the empowerment of VHVs is widely studied as a tool for improving community-level health performance, there is limited evidence on empowerment as a clinical mechanism to improve the health status of VHVs themselves. This is a significant oversight, given that national data indicates approximately 50% of Thai VHVs are themselves at high risk for NCDs, including hypertension and obesity [8].

Health Empowerment Theory (HET) posits that empowerment is a multi-level process of mobilizing personal resources to gain control over one’s health outcomes [9]. While HET has been applied to general patient populations, its utility in transforming “at-risk” community health providers—who must balance their own chronic risks with their community service roles—remains underexplored. Within the Maha Sarakham municipality, where localized screening identified a 33% prevalence of abdominal obesity, there is an urgent need to determine if theory-based promotion can translate into measurable physiological improvements. Prior research has successfully used HET frameworks to enhance health literacy and self-efficacy among community-based educators and workplace populations, demonstrating that empowerment is a viable mechanism for both primary prevention and chronic care [10]. The research specifically targeting CHWs has identified a critical need for empowerment-based interventions to address the paradox where these health mediators often struggle with their own NCD risk factors [11]. However, there remains a paucity of research applying HET specifically to frontline health volunteers who occupy a dual role as both health promoters and at-risk individuals.

This study addresses the existing knowledge gap by implementing a HET-based, personalized health promotion program targeting the health outcomes of the community workforce directly. By employing a mixed-methods approach, this research seeks to distinguish the self-care needs of at-risk volunteers from those in the general CHW empowerment literature. Guided by this framework, we hypothesized that the intervention would significantly improve the clinical profiles of at-risk volunteers through statistically significant reductions in weight, BMI, and metabolic markers. Qualitatively, we anticipated that increased self-efficacy and mastery experiences would provide an explanatory framework for these physiological changes, demonstrating a positive feedback loop between perceived empowerment and clinical outcomes.

## 2. Materials and Methods

### 2.1. Study Design

A mixed-method study (convergent parallel design) has been approved by the Ethical Review Committee for Human Research at the Maha Sarakham Provincial Public Health Office (010/2569). The study was conducted at the Maha Sarakham Public Health Service Center, a local primary care unit, beginning in August 2025. Written informed consent forms were obtained from all participants.

### 2.2. Participants and Baseline Assessments

Convenience sampling was used to recruit thirty-two VHVs. Thirty people were assigned to the intervention after two had been removed (one for not meeting the inclusion criteria and the other for refusing to participate). The inclusion criteria were good leadership, relationship-building, respectfulness, a public service mindset, and the ability to communicate effectively and provide informed consent. Exclusion criteria were having NCDs or taking any medication. These criteria are verified through a combination of the following methods: (1) Peer nomination: Since criteria like good leadership, respectfulness, and a public service mindset are subjective behavioral traits, they are verified by VHV chairpersons. The heads of the local VHV network recommend individuals who already demonstrate these qualities in their daily community work. Secondly, the health center logs from the local primary care unit (PCU) were also considered. (2) Behavioral pre-screening: The researchers conducted a brief screening interview to assess the participant’s ability to communicate effectively and to ensure the participant understood the study well enough to provide informed consent. (3) Medical screening (for exclusion): While the inclusion criteria focus on character, the exclusion criteria (NCDs or medication use) were verified through review of medical history and baseline laboratory tests.

After recruitment, the participants were evaluated for anthropometric parameters. Throughout the study, 9 participants discontinued the intervention due to relocation (*n* = 2) or personal reasons (*n* = 7). Thus, the final analysis included 21 participants. The parameters measured by the InBody body composition analyzer (InBody 770) include, among others, body water (L), protein (kg), minerals (kg), body fat (kg), weight (kg), skeletal muscle mass (kg), fat mass (kg), body mass index (kg/m^2^), percentage of body fat (%), waist to hip ratio, visceral fat level, visceral fat area (cm^2^), and impedance (Ω). A higher phase angle is generally considered better. Before the measurements, participants were instructed to obtain sufficient sleep and refrain from food and drinks for 8–10 h overnight. Clinical laboratory data were obtained from the routine health checkup database.

### 2.3. Phases of Intervention

**Figure 1:**
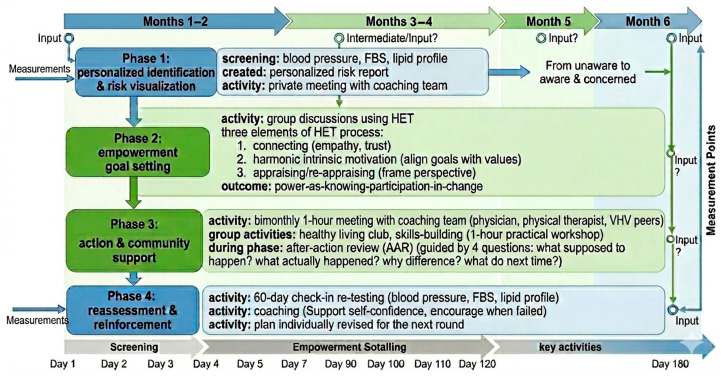
Timeline and workflow of the four-phase personalized health promotion program. FBS, fasting blood sugar; HET, Health Empowerment Theory.

The six-month personalized health promotion programs were tailored to match individual needs. It is a 4-phase, high-touch, personalized intervention designed to make invisible risks visible and empower individuals to take control (Figure 1).

#### 2.3.1. Phase 1

Personalized identification and risk visualization aimed to move the individual from unaware to aware and concerned by conducting the non-intrusive screenings for blood pressure, fasting serum glucose, and lipid profile. During this phase, the researchers created a personalized risk report for each participant and arranged a voluntary and private meeting with a coaching team.

#### 2.3.2. Phase 2

Empowerment goal setting aimed to identify personal motivations and build a plan based on the individual’s life, using HET. Based on Shearer, HET is a practice-oriented model that defines empowerment as a social process that enhances a person’s ability to meet their own needs, solve problems, and gather assets to take charge of their lives [9]. Three interrelated elements make up the process: (1) connecting (the fundamental element that creates a therapeutic relationship based on empathy, trust, and mutual respect); (2) harmonic intrinsic motivation (aligning health goals with the individual’s own values, focusing on their internal drive for change rather than imposing external goals); and (3) appraising/re-appraising (the cognitive component where the individual is guided to identify their own strengths, resources, and barriers to frame/reframe their perspective to recognize their capacity for change). This process was conducted through group discussions and empowerment, a method proven effective for NCD management [12]. The outcome of this process is “power-as-knowing-participation-in-change,” in which the individual achieves active, self-determined participation in their own health. This session, which includes a coaching team (a physician, a physical therapist, and a nutritionist) and the VHV peers, is the core of the program. To ensure intervention fidelity and consistency among the multidisciplinary coaching team, all providers (physician, physical therapist, and nutritionist) underwent a mandatory four-hour training workshop on HET prior to the study. Coaching sessions were guided by a standardized intervention manual that provided a structured framework for goal-setting. Furthermore, the team conducted weekly coordination meetings to synchronize their professional advice and ensure that all personalized health plans remained consistent with the core HET principles of intrinsic motivation and self-appraisal.

#### 2.3.3. Phase 3

Action and community support aimed at building momentum and a supportive environment. Every two months, a 1 h meeting with the coaching team was set up. The group activities included a healthy living club that facilitated sharing experiences and connected individuals with similar goals, leveraging peer support to make new social and enjoyable behaviors. It also included a skills-building session, offering a 1 h practical workshop based on the group’s identified root causes. During this phase, an after-action review (AAR) was conducted [13]. The AAR is a discussion guided by four specific, sequential questions: What was supposed to happen? (baseline, original plan, objectives, goals, and expected outcomes). What actually happened? (event timeline). Why was there a difference? (root of the analysis). What will we do next time? (development of the concrete, actionable recommendations).

#### 2.3.4. Phase 4

Reassessment and reinforcement were set to make the results of their effort visible and create a positive feedback loop. The 60-day check-in for re-testing the key numbers (blood pressure, fasting blood glucose, and lipid profile). The coaching supported their self-confidence when they were in control and encouraged them when they failed. Then the plan was individually revised for the next round.

### 2.4. Outcomes

Both quantitative and qualitative data were used to measure the success of this program. The quantitative data included anthropometric measurements, clinical chemistry, and health behavior data. The qualitative data were from a focus group discussion to assess changes in the core HET metrics (perceived control, health literacy, and self-efficacy) and the AAR (a summary of key discussion points, findings, and a list of improved actions). Three focus group discussions (FGDs), each comprising 7 participants, were conducted in a private setting at the community health center and lasted 60–90 min. A semi-structured discussion guide was used to explore themes of health visualization, intrinsic motivation, and the impact of peer support on self-care. To ensure data integrity, each FGD was audio-recorded, transcribed verbatim, and de-identified. The use of group dynamics allowed the researchers to observe how collective empowerment and mastery experiences were reinforced within the VHV peer network. The summary was distributed to the team and stakeholders for further planning.

### 2.5. Sample Size Calculation

Given an effect size of 0.926 with a justified value of 0.8 [14], an α-error level of 0.05, and a 1-β error level of 0.95, the sample size calculated using G*Power version 3.1.9.7 yields a minimum required *n* = 20.

### 2.6. Research Instruments

We employed four research tools: a health behavior questionnaire, blood chemistry, thematic analysis of focus group data, and bioelectrical impedance analysis (BIA) to measure impedance across body segments. The health behavior questionnaire was adapted from standard nutritional assessment tools and validated for the local Thai context. Content validity was confirmed by three experts, yielding an Item-Objective Congruence (IOC) index > 0.80 for all items. In a pilot test of 30 health volunteers, the tool demonstrated strong internal consistency with a Cronbach’s alpha of 0.91 [15]. These measures ensure that the instrument reliably captures the dietary and seasoning behaviors reported in this study.

#### 2.6.1. Bioelectrical Impedance Analysis

The InBody 770 touch-type electrode impedance analyzer (InBody Co., Ltd., Seoul, Republic of Korea) was used for measuring body composition. This device measures impedance across five body segments (right and left arms, trunk, right and left legs) using six different frequencies (1, 5, 50, 250, 500, and 1000 kHz) using direct segmental multi-frequency bioelectrical impedance analysis (DSM-BIA). The participants performed a standard anatomical position with their arms abducted from their torso while standing barefoot on the electrodes of the device. Total body water (TBW), extracellular water (ECW), skeletal muscle mass (SMM), and body fat mass (BFM) were among the key metrics included in the analysis. Participants had to void their bladders, fast for at least 3 h, and abstain from intense exercise for 24 h before testing to ensure measurement accuracy.

#### 2.6.2. Blood Chemistry Methods

Venous blood samples were collected for measuring glycemic control (blood sugar, hemoglobin A1c [HbA1c]), lipid profiles (total cholesterol; triglycerides; high-density lipoprotein (HDL); and low-density lipoprotein (LDL); renal function (blood urea nitrogen (BUN), creatinine, estimated glomerular filtration rate (eGFR), and liver enzymes (aspartate aminotransferase (AST) and alanine aminotransferase (ALT)). Electrolyte levels (sodium, potassium, and chloride) were also monitored to assess physiological homeostasis.

#### 2.6.3. Health Behavior Questionnaire

To evaluate the impact of the intervention on health-related lifestyle factors, including dietary habits, physical activity, and psychological stress, pre- and post-intervention assessments were performed using the 3E2S principle [16]. The nutritional behaviors were assessed using six main variables: meal frequency (three full meals daily), vegetable intake (≥4 ladles per day), fruit consumption frequency, and the use of salty or sweet seasonings. Exercise adequacy was defined as engaging in ≥150 min/week of moderate/intensive exercise [17]. Perceived stress was recorded to capture participants’ subjective psychological state.

#### 2.6.4. Thematic Analysis Method 

**Figure 2:**
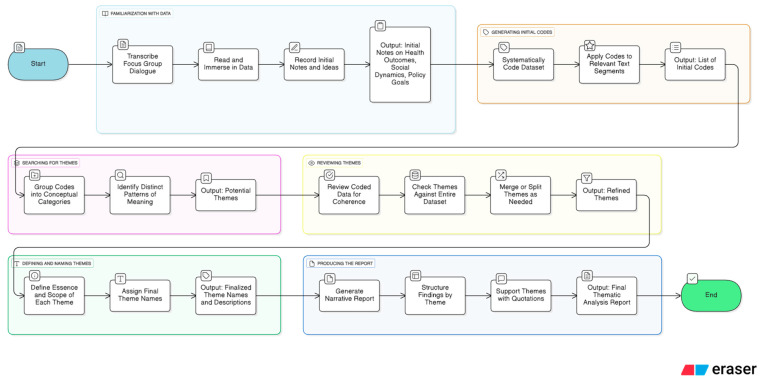
Thematic analysis workflow.

The qualitative data from the focus group interviews were analyzed using thematic analysis, following the six-phase guideline developed by Braun and Clarke [18]. This method was developed to identify, analyze, and report subtle patterns in the data, thereby capturing participants’ subjective experiences and plans. The analysis was conducted in six phases as follows: Phase 1: familiarization with the data (the researchers transcribed the raw focus group dialogue, immersed themselves in the data by repeatedly reading the text, and recorded initial notes and ideas about participants’ health outcomes, social dynamics, and policy goals). Phase 2: generating initial codes (the data were systematically coded across the entire dataset). Phase 3: searching for themes, initial codes were grouped into broader conceptual categories that captured distinct patterns of meaning. Phase 4: reviewing themes (the themes were refined in two stages: First, the coded data extracts were examined to ensure they formed a coherent pattern—internal homogeneity. Second, the themes were checked against the entire dataset to ensure they were distinct and accurately reflected the data as a whole (external heterogeneity). Themes were merged or split where necessary. Phase 5: defining and naming themes (the essence and scope of each final theme were clearly defined, detailing the specific aspects of the data each theme covered). To minimize investigator bias, initial coding was conducted independently by an external qualitative expert not involved in the intervention. Although formal inter-coder reliability (e.g., Cohen’s Kappa) was not calculated, trustworthiness was established through recursive peer debriefing. The primary authors audited candidate themes against raw transcripts to ensure findings remained authentically grounded in participant experiences. Any interpretative discrepancies were resolved through consensus-based dialogue between the coder and the research team, ensuring the final thematic framework achieved representational accuracy. Finally, phase 6: producing the report (the final narrative report was generated, utilizing the defined themes to structure the findings). The thematic analysis workflow is illustrated using the Eraser online AI tool for technical design and documentation, for illustrative purposes only, and was not used during the analytical phase (https://www.eraser.io/) (Figure 2).

### 2.7. Statistical Analyses

Statistical analyses were conducted employing IBM SPSS Statistics, version 25. The Shapiro–Wilk test was utilized to ascertain data normality. Subsequently, the nonparametric Wilcoxon signed-rank test or the χ^2^ test was used. A *p*-value below 0.05 was predetermined as the threshold for statistical significance. Data were analyzed using a Per-Protocol (PP) approach. Only participants who completed the full 12-week intervention and provided both baseline and follow-up data (*n* = 21) were included in the final analysis. Missing values resulting from participant dropout were not imputed, as the study aimed to evaluate the physiological efficacy of the completed personalized program. To assess the impact of attrition, a comparison of baseline characteristics between completers and non-completers was performed, showing no significant demographic differences.

## 3. Results

### 3.1. Demographic Characteristics

The primary patient data for this study consisted of 21 VHV leaders. The mean age was 62.43 ± 7.28 years, with 20 females (95.24%) and one male (4.76%). Overall body mass index (BMI) was >22.9 kg/m^2^, which is above the upper limit of the normal Asian BMI range [19]. More than 50% had a monthly income above the poverty cut-off point (<5000 baht) [20]. Other details are presented in Table 1.

### 3.2. Body Composition Analysis

Table 2 shows body composition parameters at the pre- and post-intervention periods. Only weight and body mass index (BMI) showed significant improvements. A significant weight loss was observed in the post-intervention period (decreased from 66.50 kg to 60.40 kg, *p* = 0.03). Similarly, BMI significantly reduced from 27.10 kg/m^2^ to 26.80 kg/m^2^ (*p* = 0.02), indicating an overall improvement in weight status after the intervention. Other body composition parameters, including body fat mass, percentage of body fat, visceral fat level, and visceral fat area, showed favorable downward trends but were not significant (*p* > 0.05). Skeletal muscle mass, segmental lean mass, and body cell mass remained stable throughout the intervention period, with no significant differences between pre- and post-intervention measurements. Additionally, basal metabolic rate (BMR) and total energy expenditure (TEE) remained stable, suggesting that the intervention did not have adverse effects on metabolic function. Changes in waist circumference, waist-to-hip ratio, or biological age were observed.

### 3.3. Blood Chemistry

Table 3 shows changes in the hematologic values between the baseline and post-intervention periods. A Wilcoxon signed-rank test was conducted to examine differences in hematologic values between the 1-month and 6-month intervention periods. Overall, the results indicated no statistically significant differences between pre- and post-intervention values across all measured variables (*p* > 0.05).

### 3.4. Health Behavior Changes

The descriptive statistics in Table 4 show changes in health behaviors over 6 months of the intervention. A Chi-square test was used to examine differences in health behaviors between the baseline and post-intervention periods. The results showed a statistically significant difference in exercise adequacy (150 min/week of moderate exercise), χ^2^(1) = 25.14, *p* < 0.001.

### 3.5. Thematic Analysis

The thematic analysis revealed four primary domains of impact, ranging from broad systemic advocacy to individual physical and social gains (Table 5). First, “Sustainability and Systemic Advocacy” (Rank 1) emerged as the most prominent theme. Health leaders expressed a strong drive to institutionalize the program, stating, “The health leaders want to develop policies for continuous activity, generating sustainable indicators to help older adults and chronic disease patients, including the general public.” Participants emphasized the importance of structural growth, noting, “I want a network to be established to help promote health and prevent chronic diseases,” and expressed a need for “publicity to increase the number of members.”

Consequently, the intervention catalyzed “Personal Transformation and Well-being” (Rank 2). The participants reported significant health improvements. For example, one stated, “My pains decreased, and I have discipline regarding eating and exercising.” These reports aligned with the InBody data, with participants noting, “I lost weight and fat, and my muscle mass increased.” As a consequence, the physical improvements might contribute to psychological improvement, as confirmed by the statement, “I have more confidence.” Furthermore, the program also facilitated the “Comprehensive Skill Acquisition” (Rank 3). The intervention moved beyond general advice to the transfer of specialized health literacy. Participants appreciated technical nutritional skills, noting, “I learned about counting carbohydrates” and “I gained more knowledge to use for proper weight control.” Furthermore, the acquisition of safe physical habits was highlighted: “I gained knowledge on correct exercise to avoid injury.”

Finally, “Social Capital and Community Cohesion” (Rank 4) was fostered through the group environment. Participants highlighted the importance of peer motivation, stating, “I was impressed that my friends saw the importance of participating in this activity.” The social aspect was a key driver of adherence, with participants mentioning, “I made more friends and had fun.” Ultimately, the program strengthened community ties, as reflected in the statement, “Friends have become more unified within the group of health leaders.”

## 4. Discussion

Village health volunteers are the cornerstone of primary healthcare in Thailand, yet they often face high personal risks for NCDs, necessitating preventive measures within this specific group to maintain their effectiveness as health leaders. This preliminary mixed-methods study was to implement and evaluate a 6-month empowerment-based, personalized health promotion program for 21 VHV leaders at high risk for NCDs. We conclude that this program is associated with improvements in selected physiological and behavioral indicators, based on the following supporting findings.

First, the BIA physiological assessment showed decreased body weight, reduced visceral fat area, and increased muscle hydration. Secondly, the clinical manifestation was non-significant but favorable downward trends in median HbA1c and LDL cholesterol, with stable organ function. Thirdly, self-behavioral assessment showed that the exercise adequacy significantly improved. Lastly, the qualitative thematic analysis identified four impact domains: sustainability/systemic advocacy, personal transformation, skill acquisition, and social capital. These findings signified that the intervention facilitated a transition from passive recipients to active health advocates. Mastery experiences—such as observing changes in physical body composition and gaining functional health literacy—were the primary drivers of increased self-efficacy. The program fostered social capital and community cohesion, both essential to collective health leadership.

Technically, BIA indicates the body’s low water content and is inversely related to muscle mass [21]. In our study, overall impedance decreased, signaling increased muscle mass. Physical activity reduced impedance, supported by improved cell membrane health and increased intracellular water (muscle hydration), as reported by Yamada and colleagues [22]. Body weight measures the total mass of all physical components and offers a snapshot of energy balance [23]. In our study, the significant overall decrease in weight reflects a measurable reduction in body mass, which aligns with the primary clinical goals of the intervention. Additionally, based on BIA data, body fat mass, body fat percentage, visceral fat area, and visceral fat mass all showed a downward trend. These results reflected a reduction in body fat percentage and indicated a positive shift in body composition, with decreased visceral fat area and mass likely contributing to metabolic health improvement. As reported by Kolb, the reduction in proinflammatory visceral fat is a known precursor to improved insulin signaling and metabolic control [24]. In our cohort, the consistent downward trends in weight and fat levels are consistent with a favorable shift in energy regulation. The reductions in body fat mass and visceral fat area are particularly noteworthy, as they suggest that participants achieved a clinically relevant change in body composition rather than just weight loss. This observation aligns with Goossens’ research, which emphasizes that optimizing fat distribution is a key factor in supporting long-term metabolic stability [25].

The results of this study also indicated that the intervention did not produce significant changes in laboratory parameters. However, several clinical positive trends were observed. The maintenance of fasting blood sugar levels and the decrease in median HbA1c values suggest that the intervention may have helped stabilize glycemic control, especially among participants with higher baseline levels. Although the reduction in HbA1c did not reach statistical significance, even modest decreases in HbA1c are considered clinically meaningful in managing chronic metabolic conditions [26].

Improvements in lipid profiles, especially the reduced total cholesterol, LDL cholesterol, and triglycerides, indicated a potential positive impact of the intervention on cardiovascular risk factors. Previous research showed that lifestyle and health-promoting interventions could produce meaningful improvements in lipid levels, even if statistical significance isn’t always reached, particularly in studies with small sample sizes or brief follow-up periods [27]. Such methodological limitations, including high interindividual variability, may explain why the present study did not yield statistically significant results.

The stability of renal and liver function parameters throughout the study indicated that the intervention was safe and not harmful to major organ systems. This aligns with previous studies showing that non-pharmacological health interventions generally do not add renal or hepatic risks when properly applied [28]. Additionally, the steady electrolyte levels suggest that overall physiological balance was maintained during the intervention.

Regarding the thematic analysis, prioritizing sustainability indicated that participants shifted from passive clients to active health advocates, reflecting modern structural advocacy in health promotion. Recent research highlights that for interventions to last beyond the study period, they must be integrated into local policy and community frameworks. Strengthening formal networks is now seen as a key factor for sustaining health programs in aging populations [29].

The second theme shows how physiological data and psychological perception align. Mentioning discipline and self-confidence aligns with social cognitive theory, which holds that achieving physical milestones serves as a mastery experience. As participants observed their body composition transition from a profile of low muscle mass and high body fat (a C-shape) to one where muscle mass surpassed body fat levels (a D-shape), their confidence in maintaining these behaviors grew, creating a positive feedback loop of well-being. The link between biomarker data and well-being is crucial for self-regulation. Recent studies revealed that mastery experiences from high-precision feedback, like InBody scans, greatly enhanced long-term adherence to diet and exercise by boosting participants’ perceived control over their behaviors [30].

The emphasis on technical skills, such as carbohydrate counting and proper exercise form, underscores the importance of health literacy in managing chronic diseases. This marks a shift from basic to functional health literacy. Modern interventions stress that technical self-management skills (e.g., accurate nutritional tracking and biomechanical awareness) are critical for patients to independently manage chronic conditions in a post-pandemic healthcare environment [31]. The rise of social capital shows that the intervention fostered a community of practice. The group integration among the participants suggests that the intervention’s social environment catalyzed collective efficacy, a measure community-based health programs [32,33].

To achieve a comprehensive understanding of the intervention’s impact, the quantitative outcomes were integrated with thematic insights from participant interviews. While the clinical data confirmed reductions in body mass and BMI, the qualitative findings provide the behavioral context for these shifts. Specifically, participants reported frequent mastery experiences—such as a newfound ability to perform daily physical tasks with less fatigue. These lived experiences of physical competence appear to have served as a primary driver for the sustained exercise adherence observed throughout the study. Furthermore, the lack of statistical significance in certain BIA parameters can be contextualized by the finding that participants prioritized functional mobility and ‘feeling healthy’ over specific clinical metrics. This suggests that in short-term interventions, psychological habit formation and perceived rebalancing of health behaviors may precede detectable physiological changes in cellular body composition. Ultimately, integrating these data streams reveals that the intervention’s success was fueled by a reinforcing loop: physical achievements enhanced psychological motivation, which in turn stabilized the behavioral patterns necessary for weight management.

Although the intervention showed positive trends, several limitations need to be acknowledged. First, the small sample size might reduce the statistical power to detect significant changes in clinical outcomes, such as HbA1c and lipid levels. Secondly, the relatively short study duration may not have been sufficient to fully capture the extent of physiological changes, especially for markers such as HbA1c, which reflect long-term glucose control. Additionally, high variability in baseline lab values and individual lifestyle habits could have masked the intervention’s effects. Relying on participant reports for behavioral data may also bring about recall bias. Finally, since this was a community-based intervention aimed at health leaders and vulnerable groups, the results may not be generalized to the broader population unless additional validation across multiple centers is conducted.

## 5. Conclusions

This research study demonstrates that an integrative health behavior program can lead to physiological changes, including improved body composition, and positive increased psychological well-being. Participants experienced a notable shift in energy balance, as evidenced by reductions in body weight and impedance, increases in muscle hydration and skeletal muscle mass, and decreases in pro-inflammatory visceral fat. Although biochemical markers, such as HbA1c and lipid profiles, showed mostly positive trends rather than statistically significant changes, the stability of kidney and liver markers confirmed the intervention’s safety. Additionally, qualitative data also indicated a shift from passive engagement to active health advocacy, with physical milestones—such as changing from a C-shape to a D-shape on InBody scans—serving as mastery experiences that boost self-efficacy. Ultimately, gaining functional health literacy and building social capital within a community fostered collective efficacy and social cohesion, supporting the long-term success of these health efforts. The practical implications of this study are significant for the Thai primary healthcare system and the management of village health volunteers (VHVs). The primary takeaway is that personal health transformation is the engine of community leadership, shifting from educators to role models. Previously, VHVs were trained to give health advice to others. This study suggests that when VHVs experience personal transformation and mastery experiences (such as seeing their visceral fat decrease on a BIA scan), they become more effective advocates. The actionable step is to prioritize the volunteers’ health before asking them to intervene in the community. Secondly, the power of visible data in empowering the use of bioelectrical impedance analysis (BIA) led to visible improvements for participants. In a community setting, abstract concepts like metabolic health are hard to grasp. The next step is to use portable diagnostic tools that provide immediate visual feedback (e.g., body composition charts), which can significantly boost participants’ self-efficacy and program adherence in rural health settings. Thirdly, the qualitative findings on social capital and systemic advocacy suggest that health programs should not just focus on individual metrics (such as LDL or weight) but also on building a supportive peer network. Lastly, early intervention is needed for at-risk providers—the study targeted VHVs with NCD risks before they developed full-blown diseases. The next step is to highlight the need for preventative screening programs specifically for healthcare workers and volunteers, ensuring the backbone of the health system remains physically capable of performing their duties.

## Figures and Tables

**Table 1:** Basic characteristics of the participants.

Characteristics	N (%) or Mean ± SD
Sex	
Male	1 (4.76)
Female	20 (95.24)
Age	
<60 years	11 (52.38)
≥60 years	10 (47.62)
Abdominal circumference (cm)	86.38 ± 10.38
Height (cm)	155.30 ± 6.07
Body mass index (kg/m^2^)	27.45 ± 5.08
Underlying diseases	
Yes	15 (71.42)
No	6 (28.57)
Annual checkup	
Yes	17 (80.95)
No	4 (19.05)
Education	
Illiterate or primary school	4 (19.05)
Secondary school or higher	17 (80.95)
Marital status	
Married	21 (100)
Single, divorced, widowed	0 (0)
Monthly family income	
<5000 THB	14 (66.67)
≥5000 THB	7 (33.33)

**Table 2:** Body composition of the participants at the pre- and post-intervention time points.

Parameter	Pre-Intervention				Post-Intervention				
	Median	IQR	Min	Max	Median	IQR	Min	Max	*p*-Value
Body water (L)	29.70	2.40	24.40	36.50	29.20	2.10	25.40	38.10	0.42
Protein (kg)	7.90	0.80	6.50	9.80	7.80	0.90	6.70	7.80	0.55
Minerals (kg)	2.80	0.40	2.40	3.40	2.70	0.30	2.40	3.50	0.93
Body Fat (kg)	24.30	11.50	11.50	46.50	22.30	8.60	12.50	46	0.21
Weight (kg)	66.50	14.20	52.10	94.00	60.40	14.60	27.70	93.20	0.03 *
Skeletal muscle mass (kg)	22.60	1.90	18.50	27.80	22.20	1.60	19.30	29	0.30
Fat mass (kg)	24.30	11.50	11.50	46.50	22.30	8.60	12.50	46	0.21
Body mass index (kg/m^2^)	27.10	5.10	17.50	40.70	26.80	4.70	18.10	40.30	0.02 *
Percentage of body fat (%)	39	8.60	20.00	49.40	37.30	8.50	22.50	49.30	0.30
Waist to hip ratio	0.95	0.14	0.75	1.10	0.92	0.10	0.75	1.07	0.25
Visceral fat level	13	5	3	17	12	3	3	17	0.26
Visceral fat area (cm^2^)	146	114	28	298	127	84	29	296	0.15
Biological age (years)	63	8	55	77	63	9	55	77	0.64
Basal metabolic rate (kcal)	1244	80	1088	2060	1228	63	1116	1489	0.25
Total energy expenditure (kcal)	1897	176	28.80	2222	1891	97	1718	2293	0.67
Body cell mass (kg)	25.70	3.50	4.50	32	25.50	2.20	22.10	33.50	0.99
Visceral fat mass (kg)	3.60	2.40	1	8.20	3.10	1.70	1.10	8.10	0.20
Obesity degree (%)	20.40	25.40	−20.30	84.90	21.70	21	−17.80	83.40	0.23
Abdominal circumference (cm)	85.50	13.70	69.40	110.60	86.30	11.10	70.60	110.10	0.22
Target weight (kg)	54.10	3.40	48.50	66.40	53.60	3.20	48.40	69.10	0.38
Weight control (kg)	−10.30	11.20	−30.60	12.70	−8.50	9.20	−30.20	11.10	0.08
Muscle control (kg)	0.00	1.20	0.00	7.60	0.00	1.80	0.00	6.90	0.29
Fat control (kg)	−11.50	11.20	−30.60	5.10	−9.90	8	−30.20	4.10	0.10
Segmental lean mass of the right arm (kg)	2.01	0.36	1.60	2.72	2.01	0.36	1.61	2.74	0.44
Segmental lean mass of the left arm (kg)	1.94	0.37	1.55	2.67	1.93	0.35	1.56	2.71	0.51
Segmental lean mass of the trunk (kg)	18.53	1.99	16.13	22.57	18.41	2.31	16.26	22.62	0.76
Segmental lean mass of the right leg (kg)	5.62	0.65	4.34	7.78	5.63	0.78	4.48	8.16	0.81
Segmental lean mass of the left leg (kg)	5.80	0.64	4.58	7.93	5.82	0.64	4.69	8.27	0.32
Impedance (Ω)	599	94	469	807	583	69	474	785	0.42

**Note:** * *p* < 0.05.

**Table 3:** Hematologic values of the participants at the pre- and post-intervention time points.

Parameter (Normal Range)	Pre-Intervention				Post-Intervention				*p*-Value
	Median	IQR	Min	Max	Median	IQR	Min	Max	
Blood sugar (70–100 mg/dL)	102	43	79	230	102	27.50	82	164	0.99
HbA1c (<5.7%)	8	4.88	5.6	13.30	7.30	5.08	5.60	13	0.48
Cholesterol (<200 mg/dL)	218	73.50	156	325	182	54	141	306	0.15
Triglyceride (<150 mg/dL)	106.5	68.25	61	567	93	91	50	611	0.252
HDL M: ≥40 mg/dL F: ≥50 mg/dL	52	15	35	73	53	15.50	32	87	0.95
LDL (<130 mg/dL)	142	54	79	230	119	42.70	73	192	0.08
BUN (10–20 mg/dL)	13	1.8	9	18	12	5	8	18	0.99
Creatinine M: 0.6–1.2 mg/dL F: 0.5–1.1 mg/dL	0.76	0.22	0.50	1.14	0.76	0.29	0.52	1.06	0.97
eGFR (>90 mL/min/1.73 m^2^)	85.85	20.42	47.50	106.80	81.90	29.10	50.95	105.90	0.81
Uric acid M: 3.4–7 mg/dL F: 2.4–6 mg/dL	6.05	1.75	3.90	8.20	5.40	1.05	4.40	8.50	0.68
AST (8–40 U/L)	23	7.50	16	47	22	7.50	16	38	0.36
ALT (0–40 IU/L)	25	16.50	5	70	22	8.50	6	35	0.75
Sodium (135–145 mmol/L)	141	2	139	142	139.50	3	135	143	0.83
Potassium (3.5–5 mmol/L)	4.2	0.70	3.60	4.70	4.05	0.53	3.60	4.94	0.76
Chloride (96–106 mmol/L)	104	2	101	105	104	2.50	101	107	0.27

**Table 4:** Health behaviors of the participants at the pre- and post-intervention time points.

Health Behavior	Pre-Intervention (N (%))	Post-Intervention (N (%))	*p*-Value
Eating 3 full meals/day			0.77
Yes	19 (90.48)	16 (76.19)	
No	3 (14.29)	5 (23.81)	
Eating 4 or more ladles of vegetables/day			0.41
No	0	0	
1–3 days/week	6 (28.57)	10 (47.62)	
3–6 days/week	8 (38.10)	5 (23.81)	
Every day	7 (33.33)	6 (28.57)	
Eating fruit 3 meals/day			0.77
No	(4.76)	0	
1–3 days/week	14 (66.67)	(66.67)	
3–6 days/week	4 (19.04)	5 (23.81)	
Everyday	2 (9.52)	2 (9.52)	
Adding salty seasonings			0.18
No	10 (47.62)	13 (61.90)	
Sometimes	9 (42.86)	8 (38.10)	
Every meal	2 (9.52)	0	
Adding sweet seasoning			0.57
No	(19.04)	7 (33.33)	
Sometimes	14 (66.67)	11 (52.38)	
Every meal	3 (14.29)	3 (14.29)	
Drink a sweet beverage			0.94
No	6 (28.57)	5 (23.81)	
1–3 days/week	14 (66.67)	14 (66.67)	
3–6 days/week	1 (4.76)	2 (9.52)	
Everyday	0	0	
Exercise adequacy (≥150 min/week of moderate exercise)			0.001 *
Yes	5 (23.80)	13 (61.90)	
No	16 (76.20)	8 (38.10)	
Perceived stress			0.29
Yes	3 (14.29)	1 (4.76)	
No	18 (85.71)	20 (95.24)	

**Note:** * *p* < 0.05.

**Table 5:** Thematic analysis of focus group data.

Rank	Theme	Supporting Dialogue
1	Sustainability and Systemic advocacy	“The health leaders want to develop policies for continuous activity, generating sustainable indicators to help older adults and chronic disease patients, including the general public.”
		“I want a network to be established to help promote health and prevent chronic diseases.”
		“I want there to be publicity to increase the number of members.”
2	Personal Transformation and Well-being	“My aches and pains have decreased, and I have discipline regarding both eating and exercising.”
		“I lost weight, lost fat, and my muscle mass increased.”
		“I have increased confidence.”
3	Comprehensive skill acquisition	“I learned about counting carbohydrates.”
		“I gained more nutritional knowledge to use for proper weight control.”
		“I gained knowledge on correct exercise to avoid injury.”
4	Social capital and Community cohesion	“I was impressed that my friends saw the importance of participating in this activity.”
		“I made more friends and had fun.”
		“Friends have become more unified within the group of health leaders.”

## Data Availability

The data presented in this study are available on request from the corresponding author due to ethical concerns and privacy protections for study participants.
